# Making bioinformatics training FAIR: the EMBL-EBI training portal

**DOI:** 10.3389/fbinf.2024.1347168

**Published:** 2024-01-31

**Authors:** A. L. Swan, A. Broadbent, P. Singh Gaur, A. Mishra, K. Gurwitz, A. Mithani, S. L. Morgan, G. Malhotra, C. Brooksbank

**Affiliations:** EMBL’s European Bioinformatics Institute (EMBL-EBI), Wellcome Genome Campus, Cambridge, United Kingdom

**Keywords:** bioinformatics training, FAIR (findable, accessible, interoperable, and reusable) principles, training website development, Agile Scrum methodology, user experience (UX) design

## Abstract

EMBL-EBI provides a broad range of training in data-driven life sciences. To improve awareness and access to training course listings and to make digital learning materials findable and simple to use, the EMBL-EBI Training website, www.ebi.ac.uk/training, was redesigned and restructured. To provide a framework for the redesign of the website, the FAIR (findable, accessible, interoperable, reusable) principles were applied to both the listings of live training courses and the presentation of on-demand training content. Each of the FAIR principles guided decisions on the choice of technology used to develop the website, including the details provided about training and the way in which training was presented. Since its release the openly accessible website has been accessed by an average of 58,492 users a month. There have also been over 12,000 unique users creating accounts since the functionality was added in March 2022, allowing these users to track their learning and record completion of training. Development of the website was completed using the Agile Scrum project management methodology and a focus on user experience. This framework continues to be used now that the website is live for the maintenance and improvement of the website, as feedback continues to be collected and further ways to make training FAIR are identified. Here, we describe the process of making EMBL-EBI’s training FAIR through the development of a new website and our experience of implementing Agile Scrum.

## 1 Introduction

As the need to analyze biological data continues to grow, so does the need for training in bioinformatics. This extends beyond formal education delivered at university level, given continual developments in methodologies, tools, and technologies used in this ever-changing field (Işık et al., 2023).

EMBL-EBI provides a broad range of training in the field of data-driven life sciences, including hands-on training for researchers to learn how to analyze their own data and training on EMBL-EBI data services to help users get started with accessing, using, and submitting data. The training spans a variety of formats including face-to-face courses on site at EMBL-EBI and at host organizations, virtual courses conducted online, webinars, and on-demand self-paced tutorials and recorded webinars, all of which are openly available on the EMBL-EBI Training website for anyone to access, anytime.

Since 2016 (Wilkinson et al., 2016), there has been a drive to make scientific data more useable and reproducible using the FAIR data principles. The FAIR principles, Findable, Accessible, Interoperable, and Reusable, revolve around clearly describing data, making data available in ways that are accessible, and using formats that allow everyone who needs access to the data. These principles can also be applied to training materials to make them more useable by both learners and educators (Garcia et al., 2020; FAIR, 2023).

In 2021, aligned with EMBL-EBI’s aim to make data FAIR, a new website was released which made EMBL-EBI’s training more FAIR. This included ensuring that information about our live courses aligned with the FAIR principles. On-demand training and materials from live courses were already being shared openly. However, users reported they were difficult to find and so focusing on findability and taking a FAIR approach led to improved ways of sharing such training.

The training available on the EMBL-EBI Training website is divided into two sections, Live and On-demand. The Live section includes all our training that happens at a specific time, including our face-to-face and virtual courses, and webinars. On-demand training features all content that is accessible to anyone, at any time through the website. This includes online tutorials, recorded webinars, course materials and, most recently, collections of on-demand content on a specific topic, and trainer pages which feature both EMBL trainers and those who join us from other companies and institutes.

## 2 Materials and equipment

**FIGURE 1:**
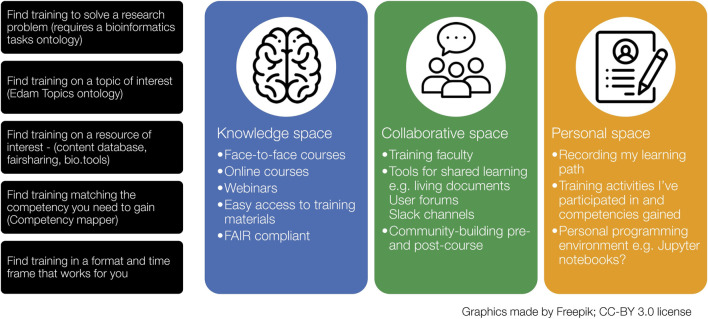
Initial vision for the new EMBL-EBI training website.

The redevelopment of the EMBL-EBI training website began with the construction and socialization of a vision for a new website that had users’ requirements and user experience at its heart (Figure 1). This vision was driven by the fact that, in 2019, the EMBL-EBI training website (actually two separate sites, one for live and one for on-demand training, with a semi-automated catalog layer on top) had evolved organically and had failed to scale adequately–either to the amount and complexity of content that we were delivering or to the user base which was already in the range of 100s of 1000s of users per year. The complexity of the two separate websites also made navigation between different types of training challenging for users and made findability of relevant content difficult, as it was not possible to search for specific topics. These requirements aligned with the FAIR principles and so applying these principles to the presentation and sharing of training and materials gave us a focus for the website’s development. There was also an additional driver to visually align the EMBL-EBI Training website with the emerging EMBL visual framework (Hawkins, 2021), an open visual standard that was being developed to provide consistency across EMBL’s family of websites. Then in 2020, after the start of the new website’s development, the COVID-19 pandemic also provided a different driver of change: our rapid shift to running courses virtually created new and urgent requirements to support learners joining courses online.

The initial phase of website development focused on user needs for short, self-paced online tutorials. Based on user research (see below), the platform to create tutorials needed to allow users to work through or be able to drop into a single page of a tutorial from search engine results. It also needed to be easy for course creators to add content, as this had previously been a bottleneck to creation and updating of tutorials.

Several different e-learning platforms and content management systems (CMS) were researched and tested, including those developed for higher education. However, we found that those specifically designed for education or e-learning were too restrictive for the user journeys required or would require a lot of training for our content creators. For example, some e-learning platforms require users to complete a course in a linear journey, rather than having the flexibility to start on the page most relevant to them and choose which other pages to move to next, or when being redirected from a search engine, they would only be able to land on the front page of a course. As we had clear evidence that only a small percentage of our users follow a linear learning path, retaining this flexibility drove the choice of technology. The main CMSs we considered were Drupal, WordPress, and Moodle. In the previous website, online tutorials were created with Drupal, which gave us experience of this CMS; however, we had found that course creators found it difficult to write and edit content in Drupal: users found it difficult to understand types of page layout to choose for different content types and found the publication process cumbersome and confusing. We also tested LearnPress, an e-learning plugin for WordPress. We did not find the setup of courses to be intuitive, and so it did not fit with our aim of making the creation of courses as simple as possible.

Based on this research, WordPress (without the LearnPress plugin) was chosen to create the online tutorials, primarily because of its flexibility and ease of use for authors. Each tutorial is a WordPress Minisite which is created and edited through one central WordPress installation. It was also possible to add the H5P plugin to the WordPress installation. H5P allows for the creation of interactive elements, such as quizzes, interactive images, and drag-and-drop elements which can be added into the online tutorials to make the content more interactive (H5P, 2023).

While WordPress was chosen to create online tutorials, to provide the required flexibility, a second CMS, Drupal, was selected to handle the information about courses (course metadata). This included creation of course pages, which describe upcoming events, and front pages of online tutorials, containing their metadata. Drupal was chosen because it is open-source, scalable and can handle a large volume of structured data. It is also highly customizable and secure (Drupal, 2023). WordPress could have been used for both functions; however, it would have required much customization, whereas Drupal has many of the required features built in.

During development of the EMBL-EBI Training website, the EMBL visual framework, which is designed to be FAIR and open, was also implemented. The Training website development also contributed to the development of the EMBL visual framework, as new visual framework elements were created to be used in the Training website (Hawkins, 2021).

## 3 Methods

### 3.1 User requirements

Before starting the website redesign, we conducted interviews with users of the previous version of the EMBL-EBI Training website. A group of seven users who attended a live course were interviewed about their experience of using the EMBL-EBI Training website. They were asked to talk a bit about themselves, their work, why they wanted to do training and if they had any previous experience of completing bioinformatics training. They were asked how they first discovered the website and were observed moving through the website searching for relevant content. They were then asked if they had any issues accessing the website and their general experience of it. Overall, users reported that there was a lot of useful content on the website although, some had difficulty finding content on a specific topic. Some users explained that they would land on specific pages within the website from a search engine while others clicked on links in the mailing list email. There was also feedback that they did not all know where to start when looking at the list of on-demand courses and they would appreciate guidance on which courses to do first. Regarding live courses, while discussing their use of the website some of the users identified the information most important to them, which included dates, title, program, and prerequisites. Several users mentioned that they did not realize that course materials from all live courses were available until they had been informed during the live course they attended. They also stated that they would not have known where to find course materials on the website if they had known they were available. From these interviews, findability was identified as a key requirement.

At the same time as the interviews, we reviewed the 33 website support emails that we had received over the previous 2 years. This identified issues regarding users not finding information about how to complete online courses, accessibility issues such as color contrast, and difficulty finding content.

Analytics showing how users interacted with the website were also reviewed. The analytics showed the different ways in which learners were accessing and using the on-demand content. Some users found an online tutorial on the website and completed it from start to finish; these users typically had a specific learning objective and used our tutorials to fulfill that objective. Other users landed on a page within a tutorial from a search engine; these users were primarily using our tutorials as a lookup service; the page of the tutorial that they landed on from their internet search answered their question and they left the website. Google Analytics helped us to review user journeys; however, understanding the journeys of online learners was not straightforward, partly as a result of the way the website was designed to allow users the ability to drop into the middle of online tutorials. Prior to development of the new website, some initial experiments were done by making minor changes to the previous website, such as reducing the amount of content on single pages, to see what impact they made to user engagement. These experiments helped identify requirements for the new website and the two distinct user journeys observed were important to consider for the website redesign. Users needed to continue to be able to land within a page of a tutorial, not requiring them to start from page one as many e-learning courses do. To do all this, the user interactions needed to remain flexible.

During the research phase, creators of the online tutorials were also interviewed. They reported that they were put off creating courses in the previous website because they considered it to be time consuming and difficult. Therefore, the choice of technology underpinning the new website needed to work for both learners and content creators.

Finally, a handful of users in the EMBL-EBI Training team need an additional layer of access to perform administrative and editorial functions, such as sharing aggregated user statistics with other teams, creating template course mini-sites and giving course authors access to them, adding new live courses to our listings, and ultimately publishing quality-checked content to the EMBL-EBI training website. We therefore also considered our own needs and bottlenecks.

These three types of users–the learner, the course author and the training administrator–were considered and consulted throughout the development process and continue to be involved now that we have moved into ‘maintenance mode’.

### 3.2 Making training materials FAIR

The FAIR principles can be applied to training materials to help in their use by learners and reuse by other educators, by ensuring their findability and providing details about how they can be accessed and reused. Findability of training was one of the main drivers of the redesign of the EMBL-EBI Training website; however, throughout the process, all the FAIR principles guided decisions regarding what types of information to include in our course descriptions (course metadata), how content is displayed to users, and the potential for reuse of training materials by others, as explained below.

### 3.3 Findability of courses

To ensure that live training can be registered for, or that online training can be used by learners, it first needs to be findable (Garcia et al., 2020). This includes findability of both the website and the courses listed within the website.

Before designing course pages, the required details about courses were first identified. The page design then focused on the display of these course metadata. These metadata were also used to help search engines identify a course page’s relevance. For live courses, each course page includes details about the format, dates, cost, contact information, target audience, learning outcomes, organizers and trainers, program, and details about how to apply or register, depending on the type of event. Online tutorial front pages include similar details, such as target audience and learning outcomes, as well as the creators, types of materials included, estimated time required, DOI, and details on how to get started. During the initial planning phase of the website redevelopment, metadata for different types of training format were identified and compared (Table 1). Previous community consensus, in addition to information from users, guided the identification of necessary metadata (Schneider et al., 2015).

**TABLE 1:** Metadata identified for different training types during initial planning of the EMBL-EBI Training website redevelopment.

Face-to-face course	Webinar/webinar recording	Online tutorial front page
Title	Title	Title
Subtitle	Subtitle	Subtitle
Start date/time	Start date/time	Date of last update
End date/time	End date/time	
Time zone	Time zone	
Duration	Length of webinar	Time to complete
Venue		
Room		
Registration opens		
Registration deadline		
Acceptance notification date		
Register interest email		
Sponsor/In association with	Sponsor/In association with	Sponsor/In association with
Provider	Provider	Provider
Participation (e.g., open application with selection)	Participation (e.g., first come, first serve)	
		Image
Contact person	Contact person	Contact email
Scientific Organizer	Organizer	
Trainer details	Trainer details	Authors
Registration fee	Registration fee	
Registration information	Registration information	
Long description (long text field)	Long description (long text field)	Subject matter overview
Target audience	Target audience	Target audience
		Technical requirements
Learning outcomes	Learning outcomes	Learning outcomes
Programme		Course contents
Learning context (e.g. face-to-face, hybrid)		
EDAM tags	EDAM tags	EDAM tags
Keywords	Keywords	Keywords
Registration link	Registration link	
Capacity	Capacity	
	Event capacity full	
	DOI	DOI
Materials link	Materials link	
	Link to recording	Link to first online tutorial page
License	License	License
Social media links	Social media links	Social media links
Language	Language	Language
Submitter	Submitter	Submitter
Additional information		

One element of metadata that applied to on-demand content only is the digital object identifier (DOI). In line with the FAIR training materials requirements of assigning a persistent identifier, all on-demand content is assigned a DOI, as it has since 2013. A persistent identifier makes the materials easier to cite and does not have the potential to change, unlike other identifiers, such as URLs (Garcia et al., 2020). DOIs can also be linked to ORCiD IDs for individuals, giving trainers more visibility and recognition for the training materials that they contribute to (ORCiD, 2023).

**FIGURE 2:**
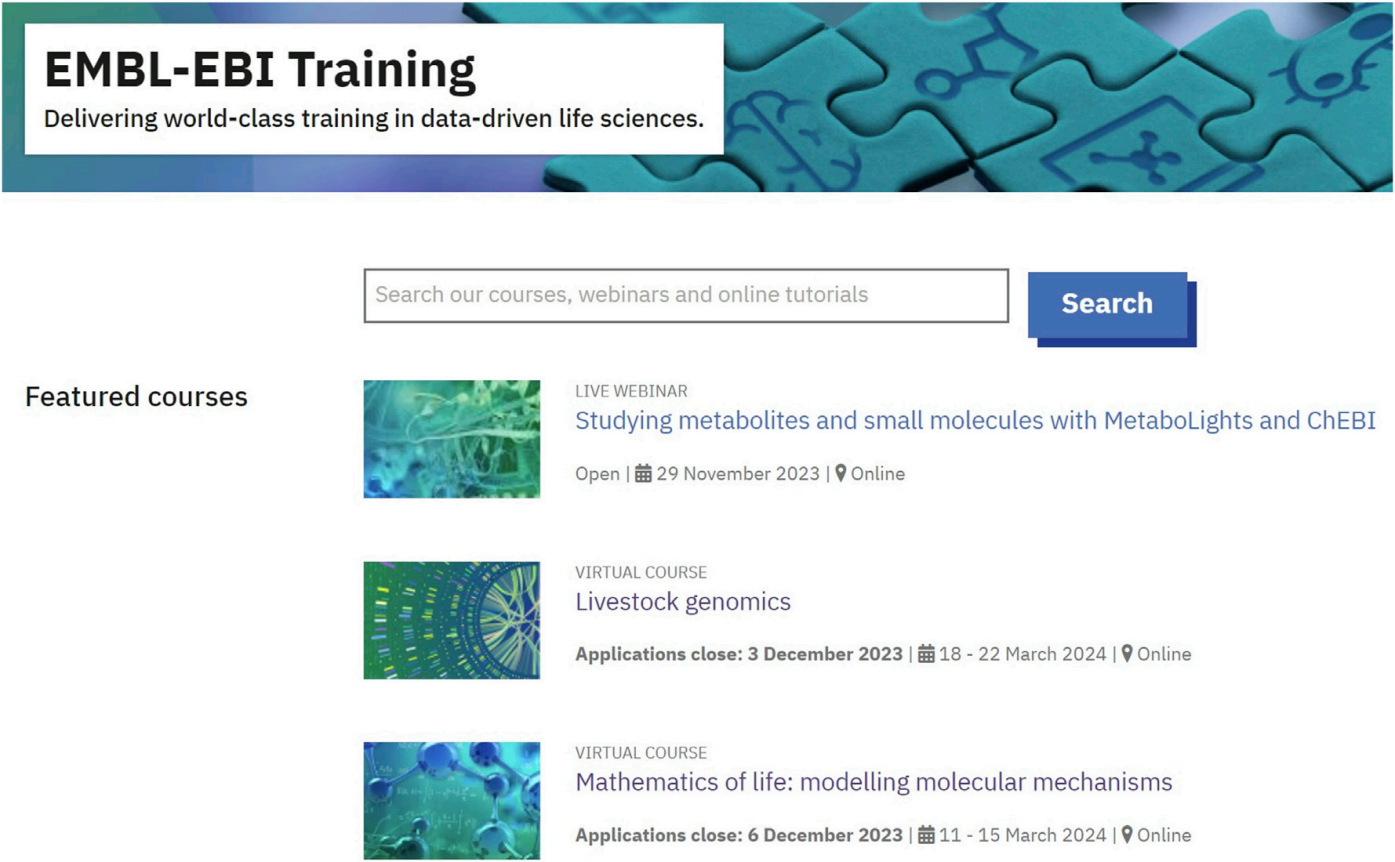
EMBL-EBI Training homepage showing search box and featured courses. The homepage can be accessed at www.ebi.ac.uk/training.

Many of the metadata listed are used to enhance the findability of courses through searching the website. A prominent search box was added at the top of the front page of the website (Figure 2). The search box uses the EBI Search functionality (Madeira et al., 2019) which allows users to search for any terms they would like whilst providing autocomplete suggestions to help users identify suitable search terms. These search terms are based on the metadata used to describe each course, including the title and description; each course is also tagged with keywords about the course topic and any EMBL-EBI data resources it relates to. Courses are also tagged with terms from EDAM, an ontology of topics relating to bioscience data analysis and management (EDAM, 2023).

To further help users find what they were looking for, filters were added both to the course listings and to the search results pages. For live courses the filters include the formats of events, application/registration status, the year it ran/will take place, and its location. On-demand training can also be filtered by format, as well as an estimated completion time.

Once a user has found training of interest, they may also wish to share it with others to help them find suitable training. Therefore, all course pages, both in the Live and in the On-demand sections, include share buttons to help users share content with others. There is also a playlist function to allow users to share a selection of on-demand training with others (see below).

To make specific courses temporarily more findable, a featured courses section was also added to the top of the homepage (Figure 2). Featured courses provide new users with a “taster menu” of EMBL-EBI courses and provide a way to highlight upcoming courses and new on-demand content.

Despite the addition of the search functionality and featured courses, feedback identified that it can still be difficult for those new to bioinformatics to know where to start; for example, some learners may not know what to search for. This prompted us to develop training Collections. Collections contain online tutorials, recorded webinars, and short challenges on a single topic to help learners get started. Example topics covered in Collections include introductory bioinformatics, exploring genetic variation, and data-driven plant sciences.

The metadata and functionality described make training more findable either by searching the website or through search engines. However, it is also important to make training more findable and to reach a wider audience by listing courses in other registries, such as EMBL’s Courses and Conferences Events listings (https://www.embl.org/events/) and ELIXIR’s TeSS (ELIXIR TeSS, 2023). To increase the accuracy and efficiency of this process an API is provided openly, and courses are detailed using the BioSchemas standard (BioSchemas, 2023). The API is read by “consumer” data listings and courses are listed accordingly. Updates to the source data are propagated to consumer course listings at differing intervals, including once a day and whenever a change is detected.

### 3.4 Accessibility

When applied to training, accessibility in the FAIR context focuses on ensuring that users have a clear understanding of how they can access training (Garcia et al., 2020). For live training, this can be achieved through a description of when and where courses are running, and how learners can apply or register. For on-demand training, accessibility describes what users need to do to access the content. This could include making it clear that there is no barrier to access, such as registration, and that users do not need to be part of a specific group to gain access.

All EMBL-EBI on-demand training is openly available; no registration or login is required to access it, including access to quizzes and other interactive content. A description of the recommended target audience is included on each on-demand course; however, it is up to the user to decide whether they fit within the target audience.

**FIGURE 3:**
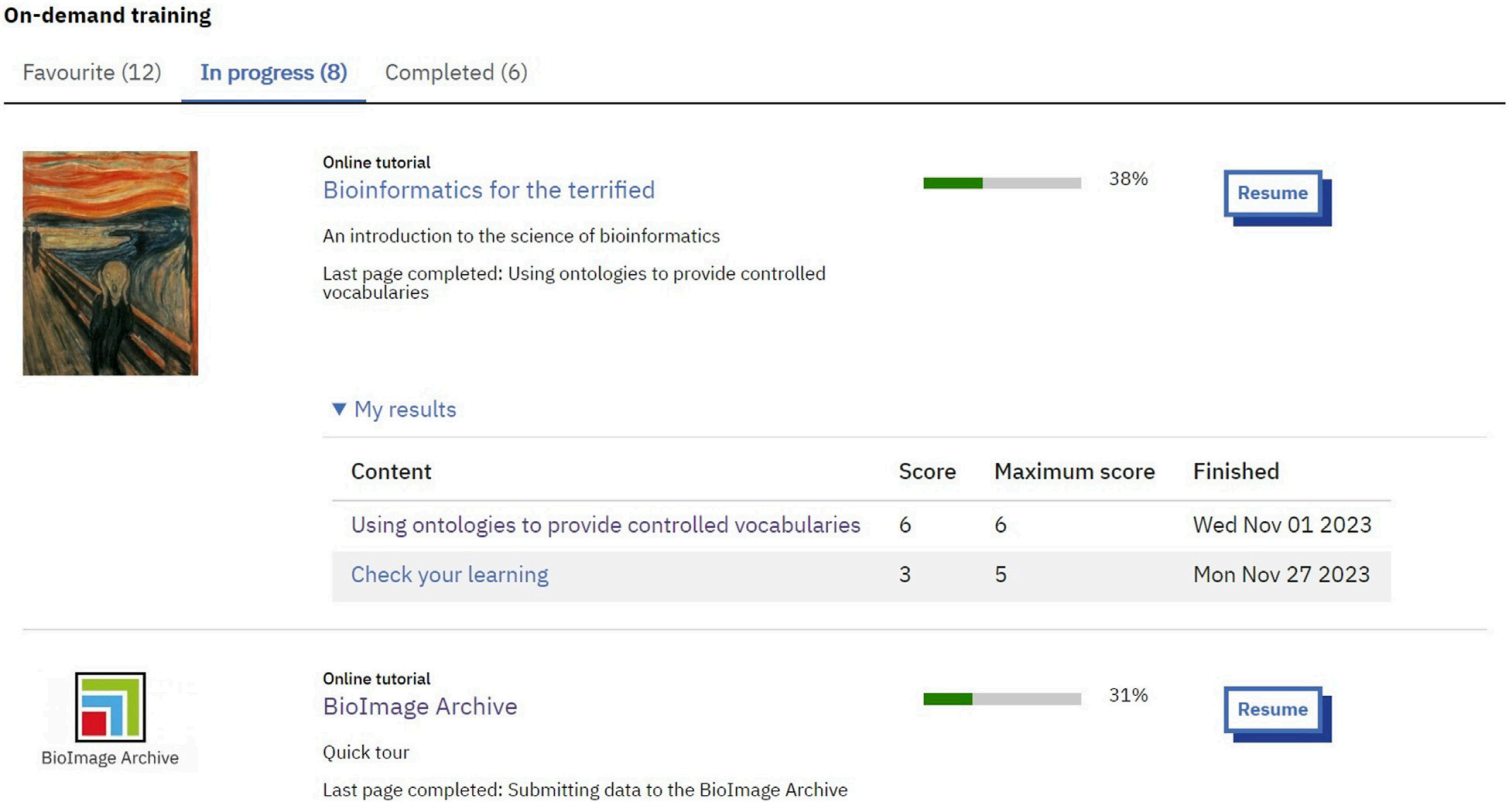
In-progress On-demand training section of a My learning page.

Nevertheless, users may choose to register for the website and log in. We added this functionality for users who would like to track their progress through on-demand training. They can mark on-demand training as favorites and see an overview of their in-progress and completed learning through a bespoke ‘My learning’ page (Figure 3). Logged in users can also create their own playlists of on-demand training, which can be shared with others, including those with no account, making it possible for learners to share suggested courses with peers, or for educators to share required learning with their students.

Attendees at our live courses are required to create an account and are given access to the course handbook through their My learning page. All information and materials required for the course are made available in the handbook. A modified version of the handbook, known as the course materials, is made available through the On-demand section following the course, so that those not able to attend may still benefit from the materials covered in the course, in a more flexible way. This significantly enhances the reach of the course materials created for our live courses–an important consideration given that (1) course materials are time consuming to produce and (2) demand for places on live courses far outstrips supply.

Throughout development of the website, accessibility of content was also considered. This included the use of alternative text for those using screen readers and reviewing color contrasts for color-blindness (FAIR training handbook, 2023). On-demand training materials are also more accessible for many compared with live training as they can be accessed anytime, from anywhere.

### 3.5 Interoperability

In the FAIR context, interoperability is concerned with the use of suitable formats and community standards that can be accessed by anyone, for example, using open-source software and open file formats in preference to proprietary software and file formats. This is directly applicable to training as providing training materials in formats that many people are not able to use makes sharing the materials useless for them (Garcia et al., 2020).

To ensure that all users can access and interact with training materials, all content, including the on-demand courses, can be used on any internet browser. This includes videos and interactive content developed using H5P. In the rare case that any other resources are required for the completion of an online tutorial it is clearly stated on the front page.

The course materials are provided in a range of file formats owing to the range of topics, tools, and trainers; however, all materials can be opened on any operating system, without the need for specialist software. Where examples and exercises are provided, trainers are encouraged to use data from open access data resources so that anyone can access both the data and the metadata to reproduce the analyses.

### 3.6 Reusability

EMBL-EBI training materials and courses are provided for people to learn from; however, they are also a key resource for other educators. Making training materials reusable by other educators avoids repeated re-creation of similar materials, saving time and effort. However, materials do need to be provided in a way that allows them to be reused providing necessary metadata, clearly describing licenses, and using modifiable formats all enhance the reusability of training materials (Garcia et al., 2020).

All the details used to describe on-demand content, such as target audience and learning outcomes, also support educators in their reuse of materials. Educators, for example university lecturers or bioinformatics trainers, are encouraged to reuse materials. Reuse could be as simple as a lecturer sharing an online tutorial with students for them to complete as part of a course, or downloading some slides initially used for a live course and repurposing them for a lecture.

For materials used during live courses, openly available sets of materials are created. These include a page per lecture or practical session with a short overview and learning outcomes to provide context. Individual files of a course’s materials are available for download, or the materials from the whole course can be downloaded from the EMBL-EBI Training FTP site.

All on-demand content also includes a release or update date so that anyone reusing the materials can be confident that they are up to date and suitable for use.

To ensure that users know what they are allowed to do with the training content made available on the EMBL-EBI Training website, the Creative Commons CC-BY license is shown on all pages of on-demand content. CC-BY allows anyone to use or modify materials in any way they would like, providing that they say where the materials came from (Commons, 2023).

**FIGURE 4:**
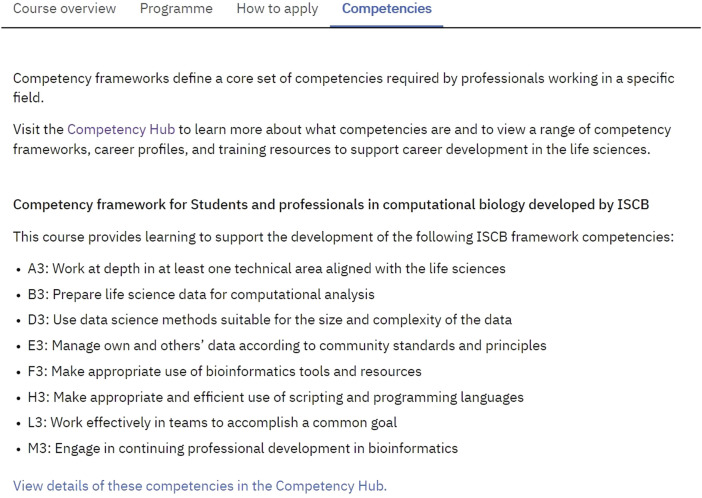
Example of competencies attributed to a training course.

To further support both learners and educators in the use of materials, additional functionality has been added to show the competencies that a learner would expect to develop by completing each course. The competencies are taken from the Competency Hub, https://competency.ebi.ac.uk/, a website that details various competency frameworks related to molecular life science and bioinformatics careers. Each framework consists of a list of competencies. Each EMBL-EBI Training course has a list of associated competencies (Figure 4). For bioinformatics competencies, the archetype is the ISCB competency framework (Mulder et al., 2018; Gaeta et al., 2021; Mendonca et al., 2022).

Applying the FAIR principles also makes the training materials we have more visible to those within EMBL. The findability of on-demand content allows for the range of topics and formats of training to be visible and potentially reused for different purposes and target audiences, and by different EMBL-EBI trainers.

### 3.7 Development process

Before starting development, a lot of planning was undertaken; the vision for a new website was socialized and the aims of the first sprints, concerned with redevelopment of online tutorials, were identified.

To identify requirement details, we first wrote user stories that outlined different types of users and their needs. We covered many different user journeys, considering the distinct requirements of learners new to the website, trainers creating content, and those who would administer the site. This process, which included web developers and user experience (UX) specialists, helped us to define what we needed to build and who we needed to build it for, rather than getting distracted by the capabilities and features of the software that we were using to build the site with. User stories define a particular type of user and summarize their requirements in terms of what they must be able to do, what they should be able to do, and what they could be able to do. The ‘must’ list provides the requirements for the minimum viable product–what is needed for a viable website to be released. If requirements from the ‘should’ and ‘could’ list were not included in the first release, they were reviewed and potentially added during a later release of the website. This allowed the development team to focus on what was strictly necessary, release early, test assumptions by soliciting feedback from users, and improve the site responsively. Examples of user stories outlining the requirements of users taking two different user journeys to the EMBL-EBI Training website are shown in Box 1; user story 1 describes a user who lands on the homepage and user story 2 describes a user who lands part-way through a course after following a link from a search engine result.

BOX 1 **Example user stories describing two different user journeys to access the EMBL-EBI Training website.**

**User story 1: potential learner/first-time visitor**

As a (potential) Learner landing on the EMBL-EBI Training homepage

I want to gain a broad overview of EMBL-EBI’s training offering

So that I can explore the content further to identify the training that best meets my needs

I must be able to:

• Recognise that I have landed on EMBL-EBI’s training “shop window”
• Be able to find relevant training in the format that best suits them
• Rapidly enter the “catalogue” to discover the scope of training on offer—in terms of topic, format, level of engagement required, and timescale
• Rapidly discover the values associated with EMBL-EBI training
• Access new and promoted content quickly
• Navigate back to the EMBL-EBI homepage
• Have links to social media accounts
• Sign up to the EMBL newsletter or our social media streams for regular updates
• Contact a person if they have questions

I should be able to:

• Learn about who EMBL-EBI’s regular trainers are on the training faculty page
• Link to the team homepage
• Tell me how I can benefit from the training on offer (knowledge, competencies)

I could be able to:

• Access testimonials from past learners
• Have a live Twitter feed/widget

**User story 2: accidental learner/fact finder**

As a user who has landed on a course page from a Google search

I want to find the fact that I was looking up and rapidly identify the broader context in which it’s set

So that I can evaluate whether there is further useful information for me in this unknown website I have just landed in

I must be able to:

• Navigate to the beginning of the course
• See my lookup content in the context of surrounding contents (i.e. view the course contents page)
• Navigate to the EMBL-EBI Training homepage from the page I landed on
• Figure out quickly that this is part of the EMBL-EBI website.

I should be able to:

• Find other content containing my lookup term, ideally ranked according to relevance.

During both the development and the (ongoing) continuous improvement phases of the EMBL-EBI Training website project, the Agile Scrum project management framework has been implemented (Rising and Janoff, 2000). This involves a small development team, with clearly defined roles, working in one- or two-week sprints to focus on a predefined set of tasks which form the Sprint Goal.

The Sprint team included Developers, a Scrum Master, and a Product Owner (Schwaber and Sutherland, 2020), drawn from members of the EMBL-EBI Training and Web development teams. Prior to starting a Sprint, the team planned which tasks to take from the Product Backlog, the list of all required tasks so far identified to develop the website, to move to the Sprint Backlog, those tasks prioritized for the upcoming Sprint.

At the end of each Sprint there was a Demo to which all Stakeholders were invited, so that they could see and provide feedback on the work completed during the Sprint and the plan for work prioritized for the next Sprint. At this stage the Stakeholders were encouraged to make comments, give feedback, or ask questions. At the end of the Sprint, the Sprint team also participated in the Sprint Review, an opportunity to identify how the team was working together, through the identification of what to continue, what to start doing and what to stop.

Throughout the development and gradual release of new website functionality, user testing was undertaken, and feedback received from learners, trainers, and other stakeholders; however, it is also important to allow for continual feedback. This is managed through email and a short feedback form that is included at the end of all online tutorials, collections and sets of materials. In the feedback form a rating is asked for, along with what the user liked about the course, what they think could be improved and if/how they plan to apply what they have learnt. This information can be used to further develop training and identify needs for future courses.

## 4 Results

The new website, developed using the FAIRification methods described, was released in February 2021 after a 3-year plus planning and development period. In the lead up to this full release, pilots and beta releases of some content types were released and amended in light of user feedback; for example, a single example of a newly designed on-demand course was released early on. Although the redevelopment project has drawn to a conclusion, continuous improvement is ongoing. A committee reviews and prioritizes tasks, and short sprints, undertaken by a more compact team, are incorporated into our schedule.

Over the first 2 years after release, an average of 58,492 users per month have accessed the site, including those who applied for live courses and users of on-demand training.

As of November 2023, the EMBL-EBI Training website contains nearly 200 recorded webinars, 81 online tutorials, nine collections, and 30 sets of course materials, as well as the live courses that are listed on the website, including both those that are open for applications and past courses going back to 2017. All follow the FAIR training principles.

Google Analytics showed that, in 2022, a total of 625,360 unique users accessed the EMBL-EBI Training website and there was a total of 2,199,866 pageviews. Many users landed on specific course pages directly from a search engine or were linked to the site from social media posts. Of these, 6.21% of visits to the website started on the website homepage; 65.5% of visits started on an online tutorial page and 16.74% started by landing directly on a live course page. The remainder of visits started by landing on course listings, search results, playlists created by users and pages about the training offered. For users who landed on the homepage, many clicked on one of the three featured courses that were listed at the time; there were on average 1,565 clicks on featured courses a month in 2022. Of the 12,599 who clicked on one of the featured courses, 9,081 were users new to the site.

After users had landed within the website there were also many searches using the EMBL-EBI Training website search box; 28,375 searches were performed in 2022. The top 10 search terms used in 2022 are shown in Table 2; they include both topics/methods in bioinformatics and the names of EMBL-EBI data resources.

**TABLE 2:** Top 10 search terms used in the EMBL-EBI Training website search box in 2022.

Search term	Frequency of search term use in 2022
Next-generation sequencing	1535
Metabolomics	1155
Cheminformatics	836
GWAS	705
Molecular modelling	637
Single cell	625
Biocuration	599
Uniprot	577
Metagenomics	495
Transcriptomics	488

Analytics also showed that the 625,360 users who accessed the website in 2022 used a range of different technologies. Table 3 provides an overview of the different types of browsers, operating systems, and devices used by those accessing the website.

**TABLE 3:** Distribution of browser, operating system, and device user by users of EMBL-EBI Training website in 2022.

Browser	Users(%)
Chrome	68.7
Safari	16.1
Edge	6.01
Firefox	5.82
Other	3.38
Operating system	
Windows	44.0
Android	22.2
Macintosh	21.1
iOS	9.72
Linux	2.72
Other	0.37
Device	
Desktop	68.7
Mobile	30.9
Tablet	0.89

In March 2022, the registration functionality was added to the EMBL-EBI Training website. Since then, 13,401 users from 167 countries have registered.

Through the voluntary short survey included at the end of each online tutorial, set of course materials and collection, learners have shared what they thought of the courses and if they have any suggested improvements. Although short surveys have been provided at the end of each online tutorial since the launch of our on-demand training in 2011, since the redesign we have received much more feedback, with a greater level of detail. An average of 106 online tutorial feedback forms are now being completed each month. From a total of 3,182 completed surveys, 67.6% rated the tutorial or collection they completed as “very good” or “excellent”. Many learners also shared how they plan to apply what they have learnt. This ranges in theme, but often includes plans to access data from a data resource, prepare data for submission to a resource, prepare for a new project they are working on, teach others or use their learning to support their career development. Examples of quotes that represent the major themes from online tutorial feedback forms are shown in Box 2.

BOX 2 **Example responses from the short survey included at the end of each online tutorial.**

**What did you like about the course?**

“I learned a lot; this type of challenge-based learning works really well for me!”

“Interactive elements and clear structure.”

“Guided examples were a great way to engage”

“The on-demand training is flexible.”

“If you're a learner then this is the right path to learn about bioinformatics.”

**What could be improved?**

“More videos of live demos.”

“More interactive tasks and quizzes.”

“More examples.”

**Will you apply what you have learnt in your research/work? If yes, please tell us how.**

“Accessing UniProt data programmatically”

“I will submit my results to the BIA [BioImage Archive] with sufficient metadata”

“In teaching Bioinformatics at College”

“I’m studying a bioinformatics course and this was a good base introduction to the subject.”

“I'm a student that wants to pursue a career in this area.”

## 5 Discussion

We have described the development of a new EMBL-EBI training website that places FAIR training principles at the forefront of the design and development process. Here we discuss both the methodology of developing the website and the results relating to users accessing the website in terms of technology, FAIR principles, and the developmental process employed.

It has been difficult to make direct comparisons between the original and new EMBL-EBI Training websites due to differing website structures and features (for example, the old website was essentially split into two websites) and so we focus here on discussion of the new website. Furthermore, when we launched the new website in beta, we continued to support the previous website; whilst this gave our users a choice of platform, there was no means of telling whether individual users had made use of both platforms.

### 5.1 Technology

At the start of the development process, we began by talking to users and reviewing website analytics to get as accurate a picture as possible of how people were using the previous version of the EMBL-EBI Training website. It was difficult to get a completely accurate picture of online user behavior as we could talk directly to only a limited number of users; furthermore, user behavior and rationale is not always clear from the analytics. This heavily impacted our choice of technology. It also drew us towards considering different types of users - those who deliberately seek out learning materials versus those that land by chance in the middle of a course whilst looking up facts. Both are entirely valid uses of our training content. It was therefore important to support both types of users. With only 6.21% of visits to the new website starting on the front page, it is clear this flexibility has been retained.

Flexibility in how users access our training has been retained by choosing two web development platforms that are not specifically designed for training or education, but that are key components of EMBL’s web development toolkit. Whilst this decision meant investing web developer time to build and maintain the bespoke platform, this approach has allowed us to use components developed for other parts of the EMBL website, contribute new elements to the EMBL visual framework (Hawkins, 2021), and make use of common infrastructure and content, including EMBL-EBI’s powerful search engine, and information about trainers and course authors who are members of EMBL personnel. The need for this flexibility was supported by the results which show that the majority of website users land directly into an online tutorial or live course page from search engine results, direct links, or social media links.

For development of online tutorials, WordPress has proved to be simple for content creators, enabling them to draft and collaboratively edit their tutorials in common word processing tools then simply copy and paste content into the platform once it has passed our editorial quality control process. As a result, more EMBL members of personnel are now actively involved in creating and regularly updating course materials.

Including the H5P plugin in the installation of WordPress opened up many possibilities for adding interactivity to online tutorials. Elements such as interactive images have been used frequently to help learners explore a data resource in a structured way from within the tutorial. Other interactive elements, such as quizzes, crosswords, and drag-and-drop questions, are helping learners to stay engaged and test their own learning (Box 2). Interactive H5P elements are created using simple forms and so content creators have been able to create interactivity, without specialist technical knowledge. This is helping us to meet user demand for more interactivity.

### 5.2 FAIR principles

Applying the FAIR principles to the presentation of course details, on-demand training, and materials provided a structure to the redesign of the EMBL-EBI Training website which focused on how users would be able to find, access and use content. Keeping this structure at the forefront of design and content decisions helped identify the detail and functionality required by users.

The first aim of using the FAIR principles to guide development of the website was to make sure the training was findable. Our training content had grown organically since the launch of the EMBL-EBI training program in 2007, and our previous platform provided little or no scope to organize training in more intuitive ways, especially given the expanding and evolving nature of the content, and no straightforward means of incorporating a free-text search engine. The results show that many users are finding training, some directly from search engine results and others through the website’s internal search. One of the main requirements for making training findable was the inclusion of search functionality, including the search box on the front page of the website and at the top of both the Live and On-demand listings. The results show that users are searching for a variety of terms relevant to the training available and that most of these searches use the suggested autocomplete keywords (Table 2). These keywords appear when users start typing their search. This helps users to understand the topics they might expect to find in our training platform and reduces issues with spelling mistakes in search terms. Nevertheless, users are not limited to searching for the keywords that have been associated with available courses. Reviewing the most popular search terms can also help us to identify gaps in the training available.

The featured courses, a short list of what’s new or recently opened for registration, was also added to the top of the front page of the website to support findability of new courses. From the analytics we have observed that featuring a new course does drive users to explore it. Feedback from users during testing also identified that featured courses help to raise awareness of the type of training and topics available on the site. This was useful to discover during testing as it highlighted the importance of covering a range of topics and training formats in the featured courses section. Comments from users who claimed not to know where to start or what topics were available also led to the development of themed collections (Box 2), selections of on-demand training on a specific topic, with a suggested learning pathway that takes the user from the more basic content through to the more advanced, or more niche applications. Feedback on these collections has shown that users have found it useful to know where to start on a topic, even if they do not complete the whole collection. Online question and answer webinars have also been run on specific collections to give learners an opportunity to ask questions that arise from working through the collections. The development of further collections is now planned.

Throughout development of the website, it was clear that keeping the on-demand training open and accessible by all was a necessity, in line with EMBL’s open science policy (https://www.embl.org/about/open-science/). However, to offer learners functionality for tracking learning it was necessary to require registration and logging in. The accessibility of content is made clear on the initial registration page where it is stated that registration is not required to access content, but instead to keep track of learning. With a small subset of users, 13,401, having registered for the site it seems clear to users that registration is not required, but instead an optional extra for those who want to track their learning, through their bespoke My learning page, a personalized learning profile that can be used to provide evidence of their learning (Figure 3).

During development, it was also important to keep interoperability of training at the forefront of how the training could be completed. The results show that users are using a range of internet browsers, operating systems, and types of devices demonstrating the importance for interoperability of training materials (Table 3). As further training materials are added to the website and new tools, data types, and file types become common in the ever-changing field of bioinformatics it will be important to keep interoperability in mind to ensure that exercises can be completed by everyone, regardless of their access to specific tools and technologies.

For re-use of training materials by other educators, it is difficult to know how many are using the materials for this purpose. There have been comments in feedback forms that show educators are repurposing materials (Box 2); however, as materials are openly available there is no requirement for educators to report on their use in this way. This makes it easier for them to be reused; however, it would be useful to receive more feedback from educators so that we can support them further and ensure that our materials are presented in a way that is effective for them. Applying the FAIR principles also provided us with an opportunity to think about how we can reuse our own materials; we were able to more easily review what on-demand training we had available and reuse it in other ways. Doing this led to the creation of Collections which make the most of materials we already have available by guiding learners to them in a format that sets training in the context of a broader topic and supports the user to work through the courses from basic principles to more advanced training. Reusability of materials is also important for EMBL’s trainers; making materials easily findable across a large organization has stimulated repurposing of existing content and reduced ‘reinvention of the wheel’.

Assigning relevant competencies to each course is a more recent addition to the EMBL-EBI Training site (Figure 4). The inclusion of competencies associated with each course is supporting users to discover the Competency Hub where they can learn more about the specific bioinformatics-related competencies and what they may want to focus their learning on to progress in their career. The competencies should also help educators who wish to use materials in their own teaching as they give a short overview of the knowledge, skills, and attitudes that they might expect their students to gain by completing a given piece of training.

### 5.3 Development process

Prior to starting the development of the website, the time spent writing user stories was extremely valuable for identifying what different users would require, the minimum requirements for releasing sections of the website, and prioritization of work. Including both web developers and UX specialists at this early stage helped us to start working together in partnership, bringing different ideas and experiences, and smoothing the way for the start of the development process. The exercise of identifying the metadata required for each type of training prior to development also helped us to ensure we covered all the required information and provided a focus to start discussing design and user experience.

The use of the Agile Scrum methodology provided a framework for a new team, comprising web developers and training practitioners, to work together on the development of the EMBL-EBI Training website.

This methodology lends itself well to the development of an online training resource, as it provides a structured way to include all stakeholders at defined steps in the process. It secured the commitment, at predefined times, from all members of the core development team, who worked together to achieve a shared goal. Those who were stakeholders but did not have the time (or the remit) to participate actively in sprints still had a well-defined and rapidly understood channel for receiving updates and providing feedback, in the form of sprint demos. Recording and open sharing of the demos within EMBL allows anyone with an interest in progress on the project to catch up. The iterative process of sprints ensures that something tangible is released and presented at the Demo at the end of each sprint. At that stage it can then be viewed and commented on by others. The release may not be visible publicly, but internal Stakeholders have the opportunity to see the results of the development process at regular intervals, allowing for required changes to be identified before a lot of time has been spent developing functionality that may not be suitable. Adhering to Scrum allowed for the developments and choices made by the sprint team to be visible to the Stakeholders and gave those Stakeholders the opportunity to provide input on the website development without being involved in the details discussed during the sprints. Working in short sprints also gave the development team the protected time to focus on the project without being required to shift focus to other work.

Initially, at the start of the development process, it was difficult to determine what was possible in terms of development in the set sprint length. However, after progressing through the first sprints and reviewing the tasks achieved, the team became adept at predicting what was possible within one sprint. Identifying sprint aims became an easier and more accurate process and items chosen for each sprint’s backlog became more achievable.

The iterative process of sprints helped us to incorporate new ways of being FAIR. As we continued with the development, new ways of making the training FAIR became evident and we were able to include these in subsequent sprint plans. This flexible, iterative process also helped us respond rapidly to changes in course delivery as a result of the COVID-19 pandemic. During this time, there were significant changes in how we delivered live courses: within 3 months of the first ‘lockdown’, our previously face-to-face courses were being delivered virtually. This had an impact on how we provided course information and training content to our live-course delegates, as we wrestled with the need to reduce live contact time (primarily to deal with the twin challenges of time-zone differences and ‘zoom fatigue’). Due to the format of sprints, including the potential for reprioritization at the start of each sprint, we were able to adapt to these changing requirements and priorities during the website development. The outcome of this was the development of our web-based course handbooks which are provided to learners in the lead up to each course and provide access to all the content that delegates need during a course. These handbooks are provided only to registered participants. After each course, public sets of course materials are derived from the course handbooks.

The Sprint Reviews were also a very important part of the sprint process, especially at the start of the project. Having a structured process to identify what was working well or to identify new ways of working together was very useful. While it was most beneficial towards the beginning of the project, it continued to be useful for ensuring the team worked well together throughout the project, especially as some members of the sprint team changed.

At frequent intervals throughout the redesign, we ran user testing to learn how others interact with the website. This was essential in understanding how new users would interact with the new functionality, whether they found it intuitive, and leading us in making improvements prior to public release.

### 5.4 Further work

Although the major restructuring and redesign of the EMBL-EBI training website is complete, websites are dynamic resources requiring ongoing maintenance and updates, and there is always more that can be done to improve the FAIRness of our training. We will continue to collect feedback from users, both trainees and trainers, to improve the website’s functionality and FAIRness. There are also plans to continue introducing further interactivity and practical exercises into the on-demand training to help learners stay engaged and gain more hands-on experience. The feedback from online tutorials and collections will continue to help improve content and identify topics and formats which are popular.

## Data Availability

The original contributions presented in the study are included in the article/supplementary material, further inquiries can be directed to the corresponding author.
